# Predicting the growth of lettuce from soil infrared reflectance spectra: the potential for crop management

**DOI:** 10.1007/s11119-020-09739-x

**Published:** 2020-08-10

**Authors:** T. S. Breure, A. E. Milne, R. Webster, S. M. Haefele, J. A. Hannam, S. Moreno-Rojas, R. Corstanje

**Affiliations:** 1 Rothamsted Research, Harpenden, AL5 2JQ UK; 2 Cranfield University, Cranfield, MK43 0AL Bedfordshire UK; 3 G’s Growers Ltd, Ely, CB7 5TZ UK

**Keywords:** IR spectroscopy, Crop growth, Fen soil, Linear mixed model, Partial least squares regression, LiDAR

## Abstract

How well could one predict the growth of a leafy crop from reflectance spectra from the soil and how might a grower manage the crop in the light of those predictions? Topsoil from two fields was sampled and analysed for various nutrients, particle-size distribution and organic carbon concentration. Crop measurements (lettuce diameter) were derived from aerial-imagery. Reflectance spectra were obtained in the laboratory from the soil in the near- and mid-infrared ranges, and these were used to predict crop performance by partial least squares regression (PLSR). Individual soil properties were also predicted from the spectra by PLSR. These estimated soil properties were used to predict lettuce diameter with a linear model (LM) and a linear mixed model (LMM): considering differences between lettuce varieties and the spatial correlation between data points. The PLSR predictions of the soil properties and lettuce diameter were close to observed values. Prediction of lettuce diameter from the estimated soil properties with the LMs gave somewhat poorer results than PLSR that used the soil spectra as predictor variables. Predictions from LMMs were more precise than those from the PLSR using soil spectra. All model predictions improved when the effects of variety were considered. Predictions from the reflectance spectra, via the estimation of soil properties, can enable growers to decide what treatments to apply to grow lettuce and how to vary their treatments within their fields to maximize the net profit from the crop.

**Electronic supplementary material:**

The online version of this article (10.1007/s11119-020-09739-x) contains supplementary material, which is available to authorized users.

## Introduction

Leafy crops such as lettuce and brassicas are important commercial crops in the UK. The value of these crops depends on quality indicators such as size and weight. Growers size their crops either by direct observation in the field or from air-borne imagery, which has become an established practice to connect crop phenotype with marketability and crop management decisions within the growing season (Bauer et al. 2019; Valente et al. 2020). Lettuces are sampled at frequent time-intervals for fresh-weight, head-weight and head-diameter which determine their market value and time of harvest. Lettuces that do not reach a desired size are not harvested; indeed, frequently large parts of fields are deemed not worth harvesting.

The growth of the lettuce can be restricted by stresses such as shortage of nutrients and water, low temperature, adverse weather, and pests and diseases. Within-field variation of lettuce growth in the United Kingdom is often a result of the soil's varied capacity to provide water and nutrients. If growers have dense information on soil variation within their fields, they are likely to be sufficiently well informed to make two main decisions. First, they should be able to recognize a priori where their crops will not reach a saleable quality and so where not to waste time and resources on production. Second, they should be equipped to decide how best to vary fertilizer and irrigation spatially (precision application) to maximize growth without applying excess of either. In both cases production would be more profitable and less harmful to the environment. Chemical analysis of soil by conventional wet chemistry is expensive and time-consuming. The densest affordable sampling in commercial conditions is one soil sample per ha (Muhammed et al. 2017). That has generally been adequate to estimate mean values and average fertilizer requirements. It is too coarse, however, for mapping the variation within individual fields in a way that enables growers to vary their applications of fertilizers and water rationally. Recent advances in reflectance spectroscopy could enable growers and their advisors to obtain affordable useful information on soil variation at resolutions sufficient for precision agriculture. However, estimated soil properties from reflectance spectra need to be sufficiently accurate to explain variance in crop performance and so inform management decisions.

The utility of near- and mid-infrared reflectance spectra from the soil to predict crop performance and aid management was investigated. One route, which avoids any issues of poor predictions of soil nutrients, is to examine the direct relation between the spectral data and the crop response. A strong relation could tell the grower where to expect good growth and where it is worth planting the lettuce (and where not to plant). It does not tell the grower which soil properties might be causing variation, however, and what action he or she should take to enhance yield. The second route is to predict soil chemical properties from the spectral data and identify which of those properties explain the variance in growth of the lettuce. This route, though less direct and perhaps less accurate because of the issues related to predicting soil nutrients, has greater potential for management; it should enable the grower to vary the management in accordance with the variation of individual soil properties that affect growth. To the authors’ knowledge, there are no studies that seek to explain variance in crop-yield metrics from soil properties estimated by reflectance spectra, however, work has been done using soil reflectance spectra directly to determine crop characteristics. These include predictions of grain yield in rice (Van Groenigen et al. 2003) and plant N uptake (Börjesson et al. 1999; Stenberg et al. 2005; Terhoeven-Urselmans 2008; Wetterlind et al. 2008).

**Fig. 1:**
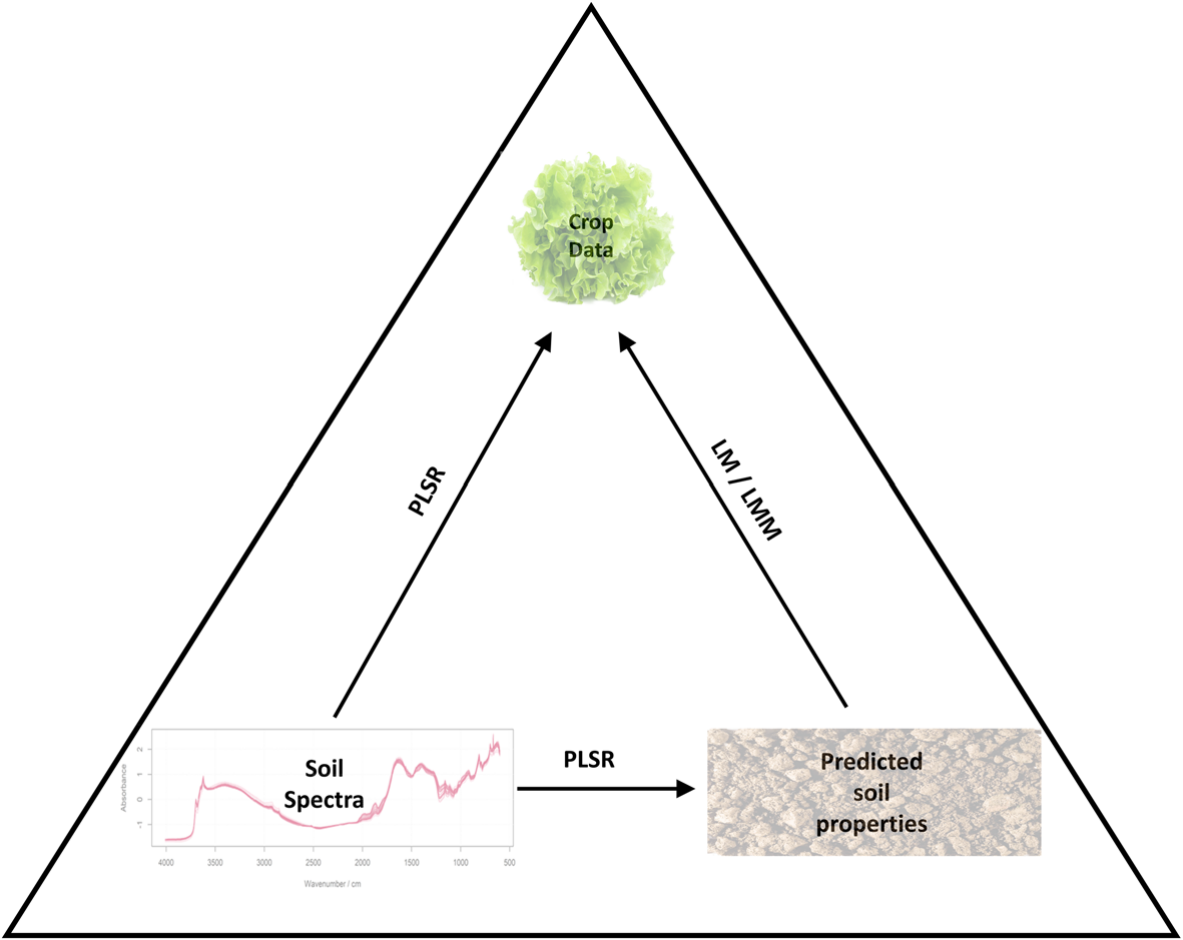
Framework for predicting crop data (lettuce diameter in cm) from soil spectra. The diameter of the lettuce may be predicted (i) directly from soil spectral measurements (the left have edge of the triangle) using partial least squares regression (PLSR) or (ii) by first predicting relevant soil properties from the soil spectra and using these in a linear model (LM) or linear mixed model (LMM) to predict lettuce diameter

This study investigated both methods of predicting crop performance from near- and mid-infrared soil reflectance spectra, i.e. directly from the soil spectra and indirectly using soil properties estimated by reflectance spectra (Fig. 1).

The following questions were addressed;

1. Can the diameters of lettuce be predicted directly from the soil-spectral data? And if so, how well?
2. How accurately can important soil properties be predicted from soil-spectral data?
3. Can values of soil properties predicted from the spectral data be used to model and predict lettuce diameter?

The results are used to discuss whether soil spectral measurements could be used in practice to help the grower (i) decide where to grow lettuce and/or (ii) manage the nutrition, irrigation or planting density of the crop.

## Methods and materials

### The fields and their sampling

The case-study is located in the Fenlands of eastern England. The soil there is generally fertile and is well suited for their growth, though within individual fields there is substantial soil variation, mainly in particle-size distribution and organic matter content. The soil on the flat land is rich in organic matter and varies from silty clay to sand on somewhat (up to 0.5 m) higher sinuous narrow strips known locally as ‘Rodhams’. It is known that the crop differs in its response on the flat land from that on the Rodhams to uniform management.

**Fig. 2:**
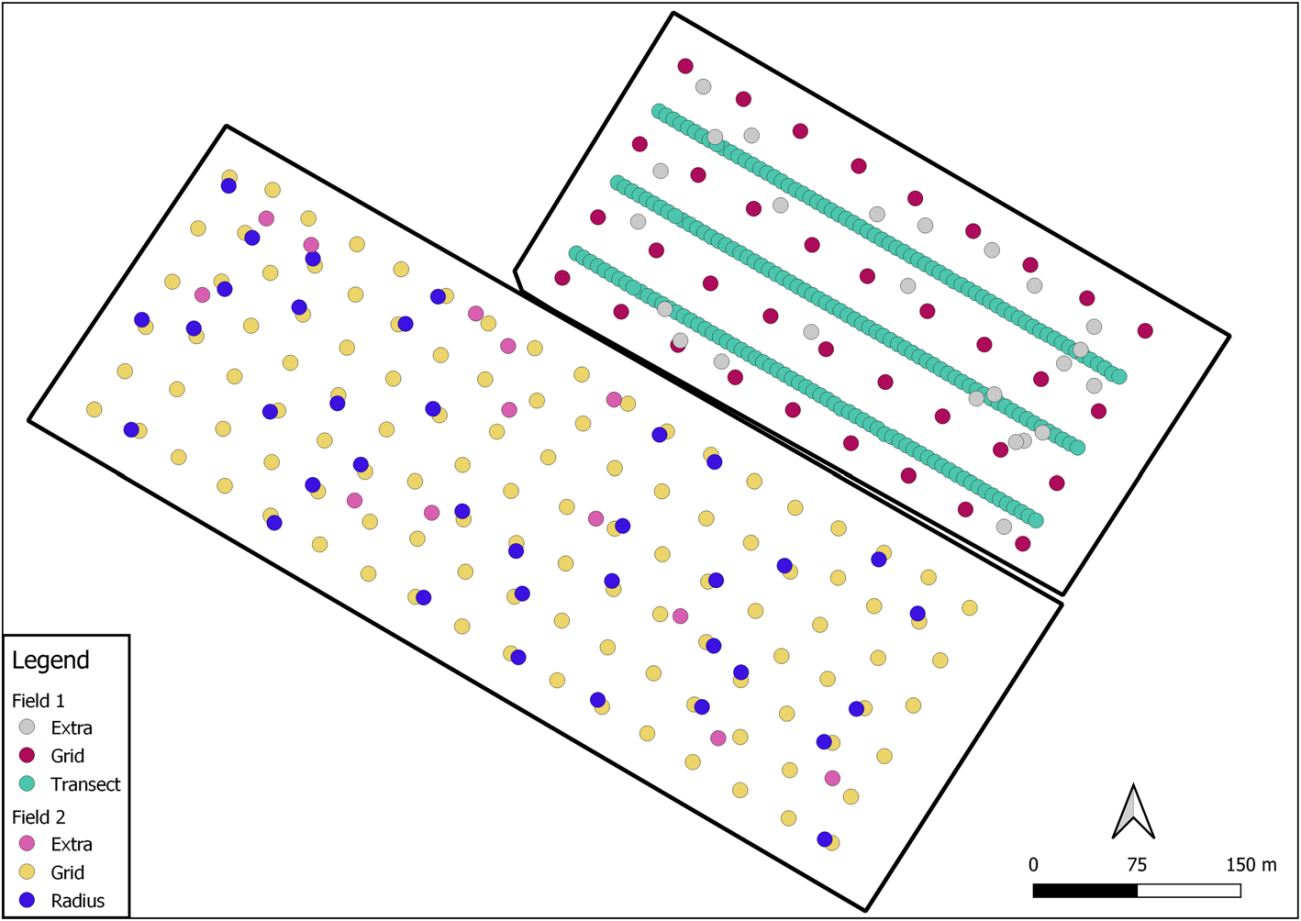
The two fields sampled. Field 1 includes the three transects sampled at 6-m intervals (Transect). The 30-m square grid (Grid) and additional sample points capture variation in soil colour and elevation from satellite imagery and LiDAR data (Extra). Field 2 includes the central points of Dirichlet tiles that all cover an equal area, leading to an approximate grid (Grid). The sub-sample of points that contains a paired sample within a 6-m radius (Radius) and additional sample points capture variation in soil colour and elevation from satellite imagery and LiDAR data (Extra)

After consulting growers in the Fenland region, two fields were chosen for this study. Field 1, covering 10.5 ha, is near the village of Prickwillow (52° 27′ 58.65″ N; 000° 21′ 51.02″ E). The sampling design was based around a 30-m square grid, with three transects (on alternate rows) more intensely sampled at 6-m intervals. The sampling strategy was designed to provide good coverage of soil conditions and to enable us to assess the magnitude and spatial scale(s) of variation in soil properties. Field 2 lies adjacent to Field 1 and covers 18.2 ha. The design was computed for 121 sample locations such that each point lay in the centre of its Dirichlet tile all of which have the same area. This was done using the spcosa package (for more detail, see Walvoort et al.  2010) which led to an approximate grid with an interval of 30 m. A further 36 of these points were selected with the BalancedSampling package (Grafström and Lisic 2019), balanced on the spatial co-ordinates and elevation. At each sample location another sample point 6 m away at a random orientation was added. The random orientation was computed with the SpatialEco package in R (Evans 2019). In both fields, additional sample locations were added to ensure that the full range of soil conditions and elevation were encompassed based on predictions from the LiDAR survey and satellite imagery showing variation in soil colour. In all, 256 samples were taken from Field 1 and 161 samples from Field 2. Figure 2 shows the fields with the sample locations.

At each sample location, three cores of topsoil (0–0.25 m) were taken within a 0.5 m by 0.5 m quadrat. These cores were bulked for laboratory analysis and spectral study.

The crop data were based on aerial imagery from which the diameter of pixel groupings that represent an individual lettuce have been calculated. There were three varieties of lettuce in each field. Aerial imagery was taken three weeks after the lettuce had been transplanted from the greenhouse, which the grower found to correspond well with the market value of the crop at harvest. The output from the aerial imagery was a polygonal shape file of circles representing lettuce diameter for each lettuce in the fields. ArcMap (ESRI 2020) was used to transform a projection for both fields based on geo-referencing the co-ordinates of individual lettuces in the polygon shape file. The mean size of lettuces within each LiDAR raster cell (0.5 $\times$ 0.5 m^2^) was calculated. Lettuce diameters were extracted for each raster cell that contained a soil sampling location giving paired soil–crop samples. Crop management in both fields followed standard commercial practice and included NPK fertilizer applications and pulley irrigation throughout the growing season.

## Laboratory measurements

### Sample preparation

The soil samples were dried in air, passed through a 2-mm sieve and milled.

The analyses on these samples are described in detail below. For spectroscopy measurements, samples were placed in a stainless-steel cup together with a disk. The samples were then milled for 35 s at 960 rpm in a TEMA Machinery Ltd mill (Northants, UK).

### Chemical analysis

Thirty sub-samples for each field were selected from the sieved samples for chemical analysis and particle-size distribution. Chemical properties and particle-size distribution of the soil samples were measured as follows.

Total carbon (C) and nitrogen (N) were determined by Dumas combustion in a TruMac Combustion Analyser from LECO Corporation (Stockport, UK).

Exchangeable potassium (K^+^), calcium (Ca^2+^), magnesium (Mg^2+^) and sodium (Na^+^) were determined in an ammonium nitrate extract (10 g of 2 mm sieved soil in 1 M ammonium nitrate) by an Optima 7300 DV Inductively Coupled Plasma-Optical Emission Spectrometer (ICP-OES) (Seer Green, UK).

Available phosphorus (P) was measured by the standard Olsen method in a sodium bicarbonate extract (5 g of 2 mm sieved soil in 0.5 M NaCO_3_ (Olsen et al. 1954) with a SANplus continuous colorimetric flow analysis from Skalar analytical BV (Breda, The Netherlands).

Sulfur (S) was measured in a potassium phosphate extract from 5 g soil in 25 ml solution. From the filtrate 9.5 ml was stabilized with 0.5 ml nitric acid ($\approx$ 68%) and analysed by ICP-OES.

The soil pH was measured in a suspension of 5 g 2-mm sieved soil to 12.5 g deionized water and measured with a thin semi-micro sealed combined pH electrode from Fisher scientific (Loughborough, UK).

Particle-size fractions were determined by laser diffraction on a L-960 particle-size analyser from Horiba scientific Ltd (Northampton, UK) in the AfSIS spectral laboratory at Rothamsted Research. The upper limit for clay was set to 9 µm since organic matter had not been removed. This is as recommended by Konert and Vandenberghe (1997) and Fisher et al. (2017). The intervals were therefore clay: < 9 µm, silt: 9–50 µm, sand: > 50 µm.

### Spectroscopy

Each sub-sample of milled soil was pressed into a small well (6 mm across and approximately 1 mm deep) and placed in a Tensor II spectrometer from Bruker scientific (Ettlingen, Germany) in the AfSIS spectral laboratory at Rothamsted Research. Its reflectance spectrum in the range 1000–2500 nm, i.e. the near infrared (NIR), was measured with a resolution of 1 nm and converted to wave-number units (cm^−1^) by division by 10^–7^. Subsequently, the moisture bands were removed in two regions: (7900–6849 cm^−1^) and (5587–5102 cm^−1^), respectively. All spectral measurements were replicated three times for each sample.

Each sub-sample's mid infrared (MIR) spectrum in the range 4000–600 cm^−1^ (2500–16 666 nm) was recorded on the same instrument with a resolution of 2 cm^−1^. The atmospheric CO_2_ bands were removed in the region 2430–2240 cm^−1^. The reflectance, $R$, in both regions have been transformed to optical density (i.e. absorbance, ${\text{A}}$) as $A = {log}_{10}(1/R)$.

Spectra were smoothed to remove noise using the Savitzky–Golay filter (Savitzky and Golay 1964) with a third-order polynomial in a moving window of 11. Subsequently, the standard normal variate was calculated (by subtracting the means and dividing the result by its standard deviation) after which the 1st derivatives of the spectra were computed. For the NIR region, a filter length of 31 wavebands was used (i.e. the spacing between points over which the derivative is computed). For the MIR region, a filter length of 11 and a segments size (i.e. the range over which the points are averaged) of 8 wavebands were used. Subsequently, the NIR and MIR spectra were combined into a single matrix used in the subsequent modelling. Processing was done using the prospectr package of Stevens and Ramiro-Lopez (2013).

## Statistical analysis

### Partial least squares regression (PLSR)

The first aim was to predict the diameters of the lettuce from the soil spectra—i.e. taking the route along the left-hand arm of the triangle in Fig. 1. Initially this was done for each field and each variety of lettuce separately. This was because the lettuce crops were grown and measured at slightly different times, and it was unknown whether the different varieties would respond differently to the soil conditions. The second aim was to predict the properties of the soil as measured by wet chemistry from the reflectance spectra. For this part of the exercise, the data from both fields together were treated as a single set. The combined data provide a wider range across the soil properties than if each field were considered separately.

Both cases are, in the general sense, a common problem in statistics: one has a set of predictor variables, ${\text{x}}\equiv \{{x}_{1},{x}_{2}...,{x}_{m}\}$, and one wishes to use the set to predict a target variable, $y$. The task might at first seem to be one of straightforward multiple regression. However, two features make that solution impracticable: (a) the spectral estimates are strongly correlated with one another, and (b) there are more of them, i.e. more variates, than there are units (lettuces). One feasible solution now popular, in such circumstances, is partial least squares regression (PLSR). This method finds a few orthogonal factors that maximize the covariance between the predictors and the target variable, or variables if there are more than one.

Let $\mathbf{X}$ be an $n\times m$ matrix of $n$ units (lettuces or quadrats) and $m$ variates (spectral estimates) and let $y$ be the vector of length $n$ of measurements (diameters of the lettuces). Then define

1 $$\begin{gathered} {\mathbf{X}} = { }{\mathbf{SL}}^{{\text{T}}} + {\mathbf{E}} \hfill \\ {\mathbf{y}} = {\mathbf{Uq}}^{{\text{T}}} + {\mathbf{f}}. \hfill \\ \end{gathered}$$

In these equations, $\mathbf{S}$ is an $n\times p$ matrix of factor scores and $\mathbf{L}$ is the corresponding orthogonal $m\times p$ matrix of loadings for the predictors in which $p\ll m$. In like manner, $\mathbf{U}$ is an $n\times p$ matrix of factor scores for the target variable and $q$ is the corresponding vector of loadings. The matrix $\mathbf{E}$ and vector $\mathbf{f}$ are error terms, which are assumed to be independent and identically distributed. These equations are solved in such a way as to maximize the covariance between $\mathbf{S}$ and $\mathbf{U}$.

In this way, the number of spectral predictors is reduced while maximizing the effectiveness of those retained. The retained components predict directly the diameters of the lettuces. In the indirect route, retained components predict the soil properties as determined by wet chemistry. The method is not quite as straightforward as multiple regression, and a final selection of the number of components retained was determined by leave-one-out cross-validation and calculation of the mean squared error (MSE) of prediction. In general, the MSE initially decreases sharply as a function of the number of components retained and then increases as a result of over-fitting. The number of components for which the MSE was least was kept. In this paper, Lin’s concordance correlation coefficient (Lin 1989) was used to get a measure of the distance from the predicted data relative to the 1:1 line. A value closer to 1 indicates a higher measure of both accuracy and precision relative to observed values.

### Multiple regression

Subsequently, linear mixed models were computed with lettuce diameter as a response and soil properties derived from the spectra by PLSR as predictor variables. Although lettuce size can be directly predicted from soil spectra, as above, those spectra do not tell growers how they might manage the land differentially to achieve some desired size of lettuce. For that, they would like to know the soil's nutrient status, carbon content and particle-size distribution. This case therefore, takes the route along the right-hand side of the triangle (Fig. 1) to answer the questions: are derived soil properties and lettuce diameters (after controlling for different lettuce varieties) related? And if so, how strong are those relations, which soil properties are deemed important for predicting the size of lettuce, and how good is the prediction along that route?

The multiple regression accounted for possible different regression coefficients for the different varieties of lettuce:

2 $$Y_{ji} = \alpha_{j} + {\varvec{\beta}}_{j}^{T} {\mathbf{x}}_{i} + \varepsilon_{i}$$

Here ${Y}_{ji}$ is the diameter of the $i$th lettuce of variety $j$, ${\alpha }_{j}$ represents the intercept for that variety, vector ${{\varvec{\upbeta}}}_{j}$ comprises the regression coefficients of the fixed effects for that variety, the soil properties are represented in the vector ${\mathbf{x}}_{i}$, and ${\varepsilon }_{i}$ is an independent residual error assumed to be drawn from a normal distribution with zero mean and variance ${\sigma }^{2}$, thus:

3 $$\varepsilon_{i} \sim N\left( {0,\sigma^{2} } \right)$$

Regression equations were set up based on this model and solved with maximum likelihood (ML) estimation. In the event, the residuals $( {\varepsilon }_{i}$) appeared to be spatially correlated with a variogram defined by

4 $$\gamma \left( {\mathbf{h}} \right) = \frac{1}{2}{\text{E}}\left[ {\left\{ {\varepsilon \left( {\mathbf{z}} \right) - \varepsilon \left( {{\mathbf{z}} + {\mathbf{h}}} \right)} \right\}^{2} } \right]$$

in which $\gamma (\mathbf{h})$ is the semivariance of $\varepsilon$ for points separated by the vector $\mathbf{h}$, with $\mathbf{z}\equiv \{{{\varvec{z}}}_{1},{{\varvec{z}}}_{2}\}$ representing the co-ordinates in the two spatial dimensions.

Most variograms of crop yields and soil properties have fairly simple forms in which $\gamma (\mathbf{h})$ increases from some small value at short separating distances to a constant or asymptote as the distance increases. To choose a suitable model, $\gamma (\mathbf{h})$ was estimated by the method of moments, thus:

5 $$\hat{\gamma }\left( {\mathbf{h}} \right) = \frac{1}{{2m\left( {\mathbf{h}} \right)}}\mathop \sum \limits_{k = 1}^{{m\left( {\mathbf{h}} \right)}} \left\{ {\varepsilon \left( {{\mathbf{z}}_{{\varvec{k}}} } \right) - \varepsilon \left( {{\mathbf{z}}_{{\varvec{k}}} + {\mathbf{h}}} \right)} \right\}^{2}$$

in which $\varepsilon ({\mathbf{z}}_{{\varvec{k}}})$ and $\varepsilon ({\mathbf{z}}_{{\varvec{k}}}+\mathbf{h})$ are the residuals at places ${\mathbf{z}}_{{\varvec{k}}}$ and ${\mathbf{z}}_{{\varvec{k}}}+\mathbf{h}$ separated by the vector $\mathbf{h}$ and for which there are $m$ paired comparisons. The variation appeared isotropic, and so the vector $\mathbf{h}$ was treated as scalar $h$ in distance only. By varying $h$ an ordered series of $\widehat{\gamma }(h)$ were obtained and these were graphed. The graphs that contained the ordered series of $\widehat{\gamma }(h)$ (experimental variograms) were used to assess whether the fitted parameter values were sensible. All could be described by the popular exponential model:

6 $$\begin{aligned} {\upgamma }\left( h \right) & = c_{0} + c_{1} \left\{ {1 - {\exp}\left( { - \frac{h}{a}} \right)} \right\} \hfill \\ & \qquad \quad {\text{for }}h > 0 \hfill \\ & \qquad \quad = 0 \hfill \\ &\qquad \quad {\text{for }}h = 0 \hfill \\ \end{aligned}$$

In this equation, ${c}_{0}$ and ${c}_{1}$ are variances, respectively, the nugget and sill of the correlated variance, and $a$ is the distance parameter. The equation describes second-order stationarity and so has equivalent covariances, $cov(h)={c}_{0}+{c}_{1}-\gamma \left(h\right)$, for incorporation into the prediction model. For convenience, model parameters were designated by $\theta \equiv \{{c}_{0},{c}_{1},a\}$. To account for the spatial correlation of the residuals ${\varepsilon }_{i}$ is replaced by ${\eta }_{i}$, which is drawn from a variance–covariance matrix $\Xi$ of error variables, i.e.

7 $$\eta_{i} { }\sim {\text{ N}}\left( {0,{\Xi }\user2{ }} \right)$$

and depends on the spatial co-ordinates of the lettuce $i$; it is now spatially dependent.

The problem now is to estimate both the coefficients of the fixed effects, i.e. the ${\alpha }_{j}$ and ${{\varvec{\upbeta}}}_{j}$, and the parameters of the random terms in ${\varvec{\uptheta}}$. This was done by the method of residual maximum likelihood (REML) introduced by Patterson and Thompson (1971) and described in the current context by Lark and Cullis (2004). Briefly, the equation that has to be solved is

8 $${\hat{\mathbf{\beta }}} = ({\mathbf{X}}^{T} {\Xi }\user2{ }{\mathbf{X}})^{ - 1} {\mathbf{X}}^{{\varvec{T}}} {\Xi }^{ - 1} {\mathbf{y}}$$

in which the matrix $\mathbf{X}$ contains all the data for the predictors plus a column of 1 s and ${\varvec{\upbeta}}$ includes the intercepts of equations (Eq. 2), and vector $\mathbf{y}$ contains the corresponding measured diameters of the lettuces. The elements of matrix $\mathsf{\Xi }$ are obtained by maximization of the likelihood

9 $$L({{\varvec{\upbeta}}}, {{\varvec{\uptheta}}} |{\mathbf{y}}) = c - \frac{1}{2}{\ln}\left| {\Xi } \right| - \frac{1}{2}\left( {{\mathbf{y}} - {{\varvec{\upbeta}}}^{{\varvec{T}}} {\mathbf{X}}} \right){\Xi }^{ - 1} \left( {{\mathbf{y}} - {{\varvec{\upbeta}}}^{{\varvec{T}}} {\mathbf{X}}} \right)$$

where $c$ is a constant.

### Selection of soil variables

Many soil properties can affect the growth of crop plants. Some of them are typically strongly correlated with one another, and to include all in regression equations could lead to spurious results from over-fitting. To avoid such an outcome, soil properties were selected that are most likely to affect the growth of lettuce. These included first the concentrations of the nutrients N, P and K. Total C was omitted as it was directly related to total N, having been measured on the same instrument. Particle-size distribution is important because it is closely related to the soil's capacity to hold water, so its measurement can serve as a proxy for water-holding capacity. The soil's pH can be important, and so it was added to the list of predictors. Finally, Mg was added because many crops suffer magnesium deficiency in the UK. The final list was as follows.

1. Total N, exchangeable K^+^, Olsen P.
2. Particle size, pH.
3. Exchangeable Mg^2+^, Ca^2+^ and Na^+^ available S.

These variables were added one at a time in the regression in that specific order (forward selection) as fixed effects using maximum likelihood (ML), since the residual likelihood is a direct function of the number of fixed effects within the model. The log-likelihood of models fitted by REML with different fixed effects are not comparable, because REML takes account of the number of parameters estimated, losing one degree of freedom for each. As the properties were added in this stepwise fashion, the updated model was tested against the previous one by a log-likelihood ratio test (Woolf 1957). A chi-squared $p$-value of 0.05 was taken as threshold and any smaller value $(p<0.05)$ as evidence that an additional coefficient explained sufficiently more of the total variance to justify inclusion of that property. Once a final set of soil properties were selected as coefficients, the model was refitted by solving Eq. 8 using REML for unbiased estimates of variance and covariance parameters. The modelling procedure described above was computed for each field individually. The partial leas squares regression was done using the pls package (Mevik et al. 2019). The linear models were computed using the nlme package in R (Pinheiro et al. 2019). Linear mixed models and the spatial correlation structures were computed using the geoR package (Ribeiro and Diggle 2018).

## Results

### Summary statistics and qualitative description of the data

Three sampling locations in Field 1 and two in Field 2 had lettuce diameters less than 50 mm and were evidently outliers. These individuals seem to have failed to establish after transplantation and were therefore removed before analysing the data formally. Tables 1 and 2 summarise the statistics of the crops with outliers excluded. Most of the measured soil properties had near-normal distributions. The pH and P were somewhat skewed, but as residuals from the PLSR were approximately normally distributed for pH and P these were not transformed. The distribution of available S and that of its residuals, on the other hand, were strongly skewed, and its concentrations were transformed to logarithms (Table 3).

**Table 1:** The summary statistics of observed and predicted lettuce diameters for *n* lettuce, along with the number of components used in the prediction, the mean squared error (MSE) and Lin’s concordance correlation coefficient (CCC) of the leave-one-out (LOO) prediction for that number of components—Field 1

Variety	*n*	Observed			Predicted			Nr comp	MSE	CCC
		Mean	Std dev	Range	Mean	Std dev	Range			
All varieties	218	14.3	3.49	15.5	14.3	2.45	15.7	7	6.15	0.66
Etude	74	12.9	3.63	15.5	12.9	3.09	15.3	4	3.59	0.84
Challenge	71	13.8	2.67	10.5	13.8	2.06	8.34	5	2.87	0.74
Glassica	73	16.2	3.24	14	16.2	2.29	10.1	4	5.23	0.66

**Table 2:** The summary statistics of observed and predicted lettuce diameters for *n* lettuce, along with the number of components used in the prediction, the mean squared error (MSE) and Lin’s concordance correlation coefficient (CCC) of the leave-one-out (LOO) prediction for that number of components—Field 2

Variety	*n*	Observed			Predicted			Nr comp	MSE	CCC
		Mean	Std dev	Range	Mean	Std dev	Range			
All varieties	106	20.6	5.01	27.3	20.6	3.51	15	5	12.6	0.66
Challenge	59	19.1	4.18	18.8	19.1	2.90	10.4	1	8.93	0.65
Glassica	26	19.6	3.47	13.5	19.6	2.39	7.58	1	6.11	0.64
Yucaipa	21	26.1	5.11	18.7	26.1	4.89	18.2	6	2.07	0.96

**Table 3:** The summary statistics of observed and predicted soil wet chemistry data for the 60 calibration samples, along with the number of components used in the prediction, the residual mean squared error (RMSE) and Lin’s concordance correlation coefficient (CCC) of the leave-one-out (LOO) prediction for that number of components

Variety	Observed				Predicted			Nr comp	RMSE	CCC
	Mean	Std dev	Range	Skew	Mean	Std dev	Range			
Total C (%)	12.31	3.67	14.2	0.39	12.3	3.64	13.9	6	0.42	0.99
Total N (%)	0.84	0.25	0.96	0.28	0.84	0.25	0.96	6	0.026	0.99
Ca^2+^ (mg kg^−1^)	7271	1137	4958	0.16	7271	1063	3786	1	400	0.93
K^+^ (mg kg^−1^)	347	112	514	1.09	347	98	487	9	54	0.87
Mg^2+^ (mg kg^−1^)	419	117	480	0.50	419	113	485	10	29	0.97
Na^+^ (mg kg^−1^)	48.8	21.4	86.6	1.09	48.8	19.5	76.9	6	8.6	0.91
Mn^2+^ (mg kg^−1^)	0.51	0.34	1.65	0.82	0.52	0.32	1.18	5	0.12	0.94
P (mg kg^−1^)	41.5	11	61.4	1.28	41.5	10.5	61.6	11	3.2	0.95
S (mg kg^−1^)	13.3	13.8	69.1	2.11	12.3	11.4	67	6	6.6	0.86
pH	7.09	0.58	2.26	− 1.37	7.09	0.58	2.31	12	0.038	1
Sand (%)	30.6	5.13	25.1	0.88	30.6	4.83	21.7	4	1.7	0.94
Clay (%)	36	4.06	209.7	− 0.74	36	3.83	17.2	4	1.3	0.94
Silt (%)	33.3	2.19	9.35	− 0.06	33.3	1.81	7.23	3	1.2	0.81

The spectral signatures of all soil samples were similar; all had smaller absorbance features in the near-infrared (NIR) than in the mid-infrared region. The NIR includes predominantly weak overtones and fundamental vibrational bands for H–N, H–C and O–H bonds. Absorption bands within the near infrared (NIR) frequently overlap, which makes it difficult to interpret the spectra directly. The MIR is characterized by fundamental frequencies (no overlap) and directly relates to mineral and organic compounds. For example, kaolinite in the Si–O stretching region between 1200 and 1000 cm^−1^ and carbon functional groups in C–H aliphatic bonds (e.g. –CH, –CH_2_ between 3000 and 2850 cm^−1^ (Viscarra Rossel et al. 2006; Du and Zhou 2009; Viscarra Rossel et al. 2011; Du et al. 2015). Loadings of the PLSR decomposition can be used for qualitative interpretation of the NIR and MIR spectra. Peaks are caused by the response variable, whereas troughs indicate interference of different soil components (Haaland and Thomas 1988).

### PLSR diagnostics

The appropriate number of components for predicting lettuce diameter and soil properties varied substantially according to variety and target property (Fig. S1 in supplementary material). For example, the MSEs of K^+^ and clay increased with numbers of components retained. This result is likely to be caused by over fitting. The MSE for P diminished gradually to a minimum at 11 components, whereas the MSE for total N diminished rapidly to a minimum at four. Tables 1, 2 and 3 report the chosen numbers of components.

**Fig. 3:**
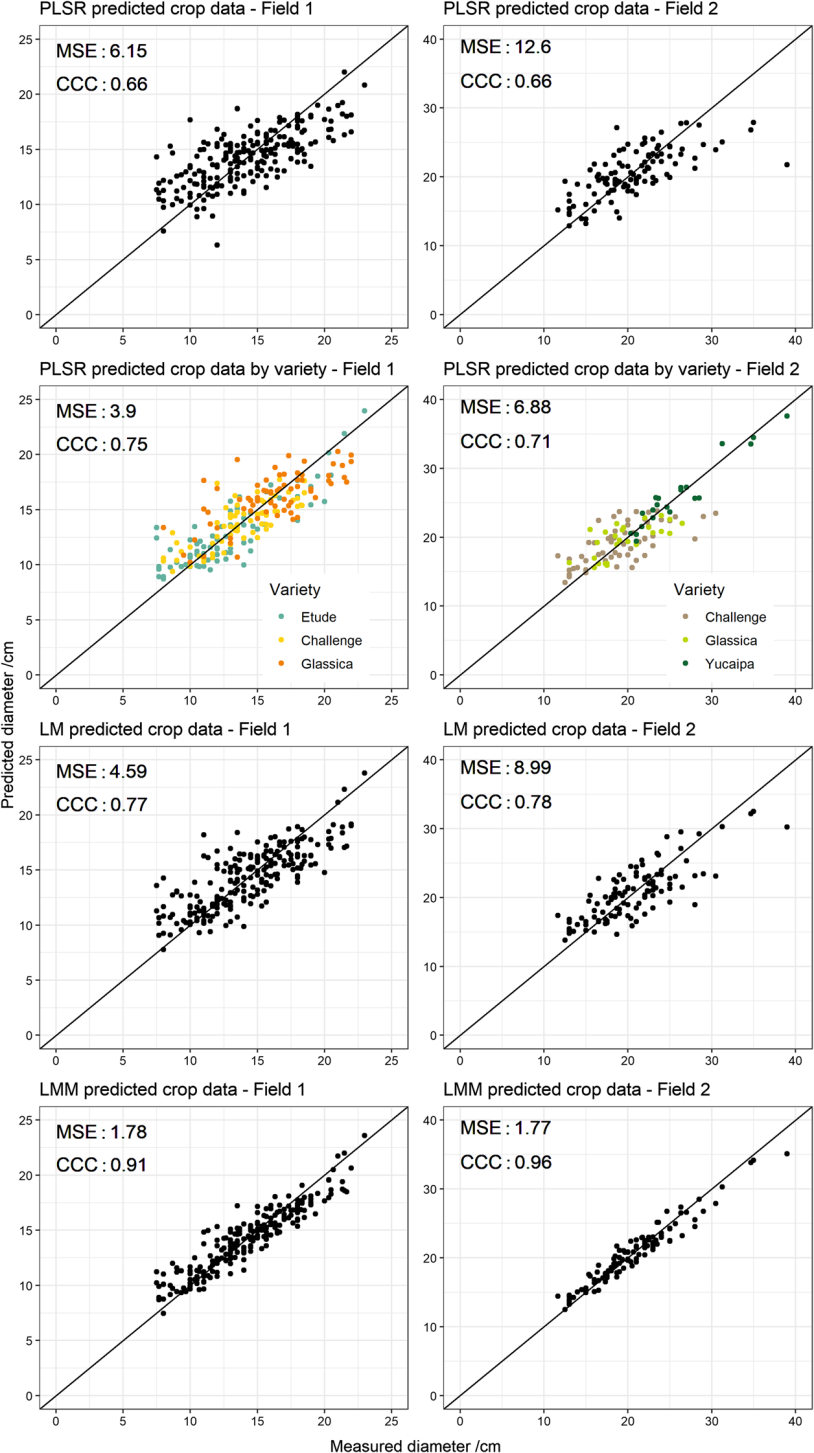
Predicted versus measured lettuce diameter for both fields. The top two rows show predictions from partial least squares regression (PLSR) from the soil spectra, where the second row takes lettuce variety into account. The bottom two rows show predictions from the multiple regression models (LM) and the linear mixed models (LMM) from the IR predicted soil properties

**Fig. 4:**
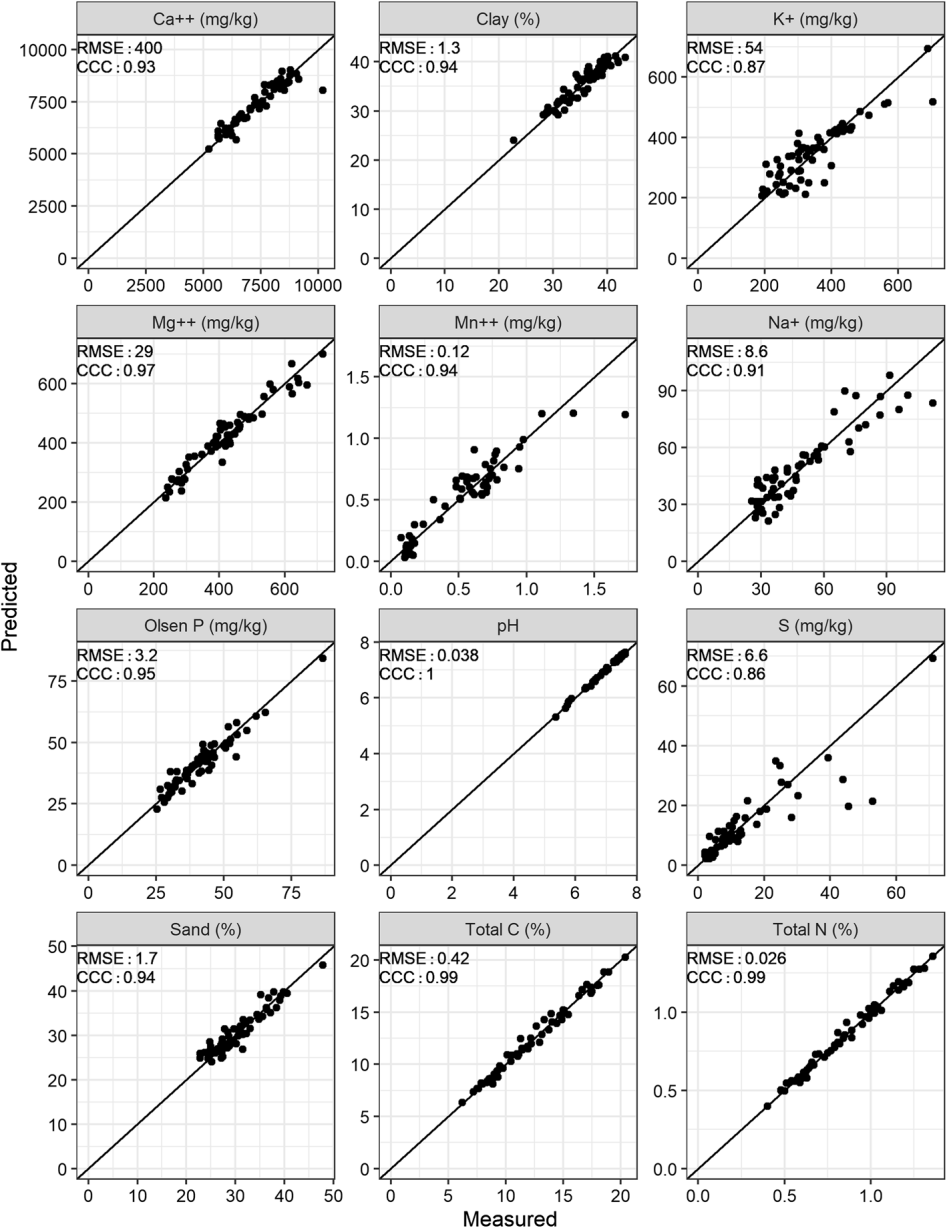
Leave-one-out predicted against measured soil properties for the 60 calibration samples from both fields by partial least squares regression (PLSR) with the soil spectra. Metrics include the root mean squared error (RMSE) and Lin’s concordance correlation coefficient (CCC)

As expected, the mean of the predicted lettuce diameters was close to the observed diameters; the standard deviations of the predictions were smaller than that of the observations. The scatter plots of predictions against the measured diameters (top 4 panels Fig. 3) show that different varieties relate differently to the soil spectra. The predictions of soil properties were generally close to true values (Fig. 4). The log ratio of sand and clay over silt were computed to provide two independent variables of particle size. The means of the predictions were also close to the observations, with again standard deviations of the predictions smaller than those of the observations. The predictions of Mg^2+^, P and pH had larger ranges than the observed ranges. Nevertheless, these differences are small and fall within the range of the residual mean squared error, leading to a slight over-prediction.

### Relations between lettuce diameter and predicted soil properties

**Fig. 5:**
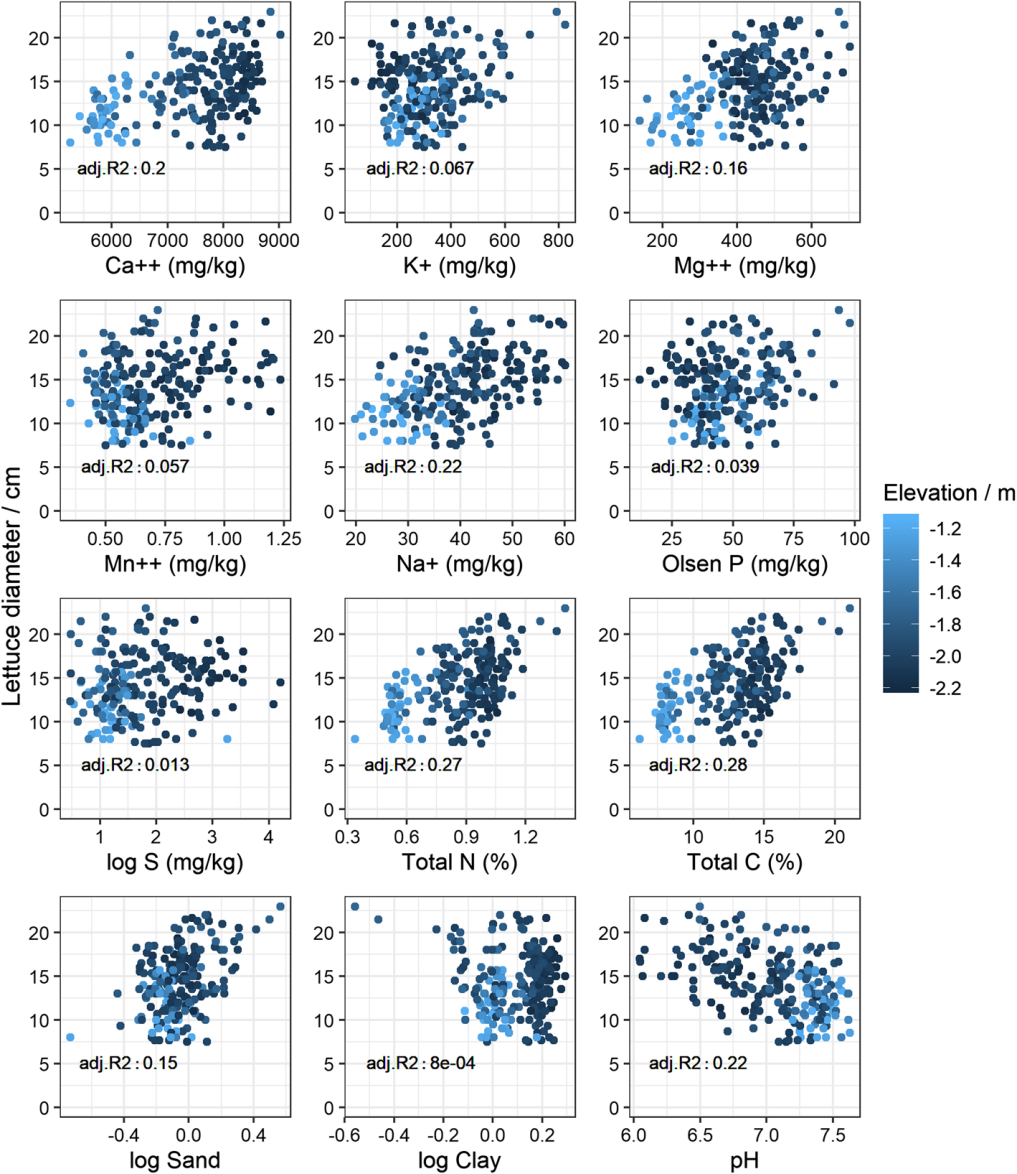
Measured lettuce diameter coloured by elevation (LiDAR) against partial least squares regression (PLSR) predicted soil properties—Field 1. Adj. R^2^ is the coefficient of determination from linear regression between lettuce diameter and the PLSR predicted soil property, adjusted by the degrees of freedom

**Fig. 6:**
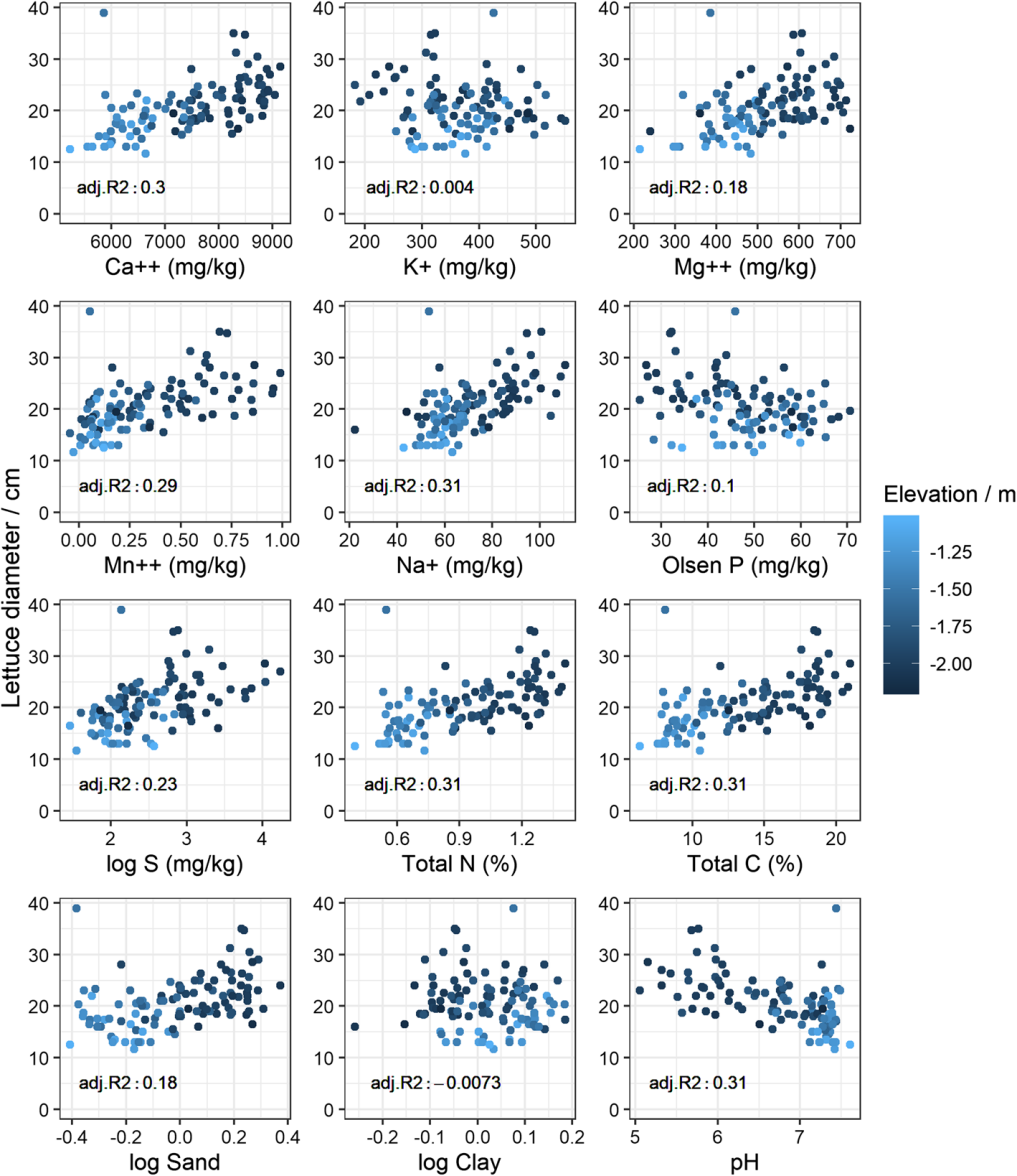
Measured lettuce diameter coloured by elevation (LiDAR) against partial least squares regression (PLSR) predicted soil properties—Field 2. Adj. R^2^ is the coefficient of determination from linear regression between lettuce diameter and the PLSR predicted soil property, adjusted by the degrees of freedom

The diameters of the lettuce were related positively to Ca^2+^, Mg^2+^, Na^+^, total N and total C in both fields and for all varieties (Figs. 5, 6). There were consistent negative relations with pH in both fields, probably because there are patches of relatively acid peat in the fields. The correlation coefficient between pH and total C was − 0.83 based on the wet chemistry measurements (not shown). In Field 2, lettuce diameter related positively to Mn^2+^, available S and to $log$(sand/silt). The relation between lettuce diameter and P was weakly negative, possibly due to a bias from the PLSR predictions. As expected from the soil-forming history in the region, the soil properties appear to co-vary with elevation (see Figs. 5, 6). The final fitted LM (Eq. 2) relating the predicted soil variables to lettuce diameter comprised total N, K^+^$(p\le 0.0001)$, $log$(clay) $(p\le 0.0001)$ and pH $(p=0.003)$ for Field 1 as coefficients; and variety, total N, K^+^ $(p=0.0187)$ and P $(p=0.001)$ for Field 2.

Further investigation of the LM residuals suggested that the exponential variogram model (Eq. 6) would describe their spatial autocorrelation well in both fields. This model was therefore included in the regression (Eq. 2). The final model for Field 1 retained variety, total N, K^+^ $(p=0.0114)$ and $log$(clay) $(p=0.0004)$ as coefficients. The final model for Field 2 retained variety, total N, K^+^ $(p=0.0195)$ and P $(p=0.0013)$ as coefficients (See Tables S1 and S2 in supplementary material for fixed effects coefficients).

**Fig. 7:**
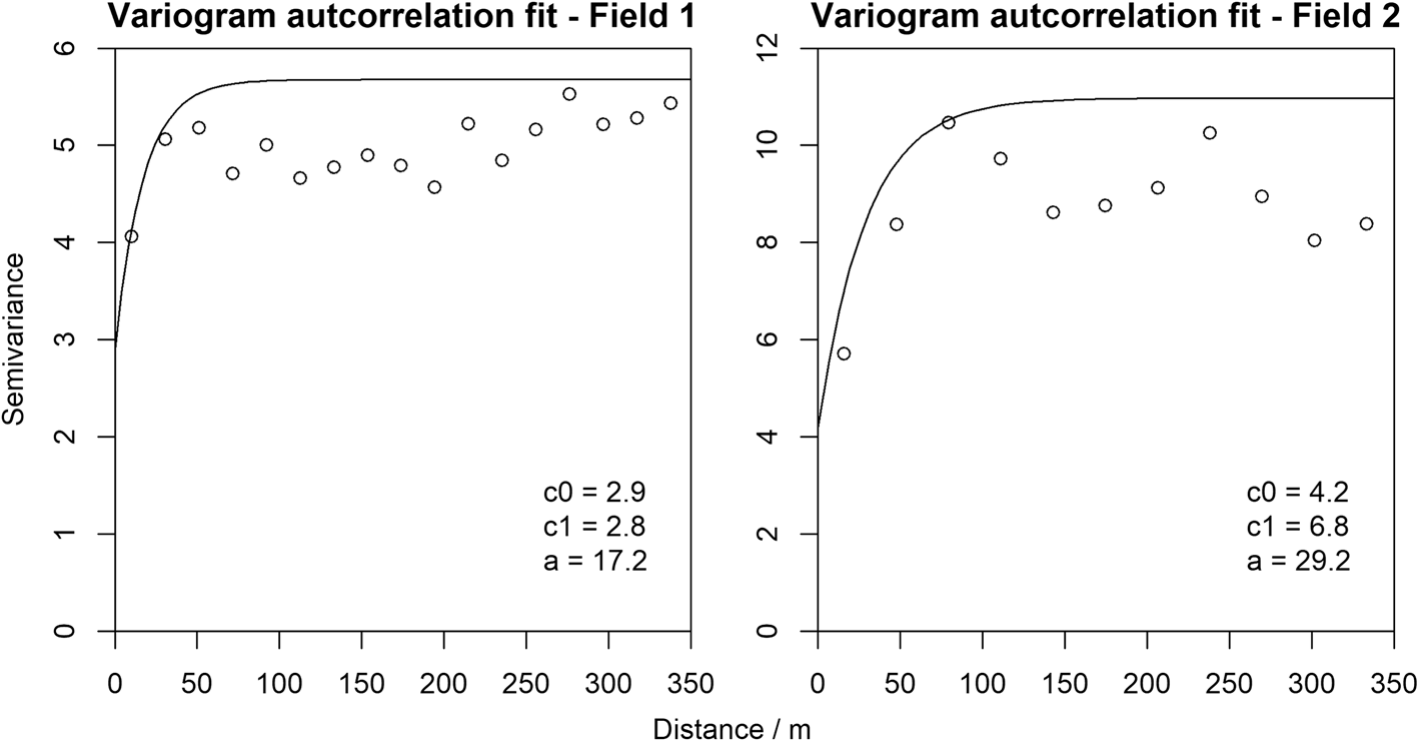
Fitted exponential model for the spatial autocorrelation term in the linear mixed model (LMM) for Fields 1 and 2, where ‘c0’ and ‘c1’ are the nugget and sill of the correlated variance and ‘a’ is the distance parameter

The variograms associated with covariance models for the spatial autocorrelation term for Eq. 6 are in Fig. 7. The parameters for Field 1 are $${c}_{0}=2.9$$, $${c}_{1}=2.8$$ and $$a=17.2$$ m. Those for Field 2 are $${c}_{0}=4.2$$, $${c}_{1}=6.8$$ and $$a=29.2$$ m. The nugget variance ($${c}_{0}$$) in Field 1 is approximately half of the total variance $${(c}_{0}+{c}_{1})$$. For Field 2, it comprises somewhat less than half of the total. The nugget includes both measurement error and very short-range, unresolved, spatial variation. The effective limit of spatial correlation, i.e. the effective range (approx $$3a)$$, in Field 2 is almost twice that in Field 1.

The mean-squared errors and Lin’s concordance correlation coefficients for the LM and LMM predictions were computed (bottom 4 panels Fig. 3).

### PLSR loadings and their interpretation

**Fig. 8:**
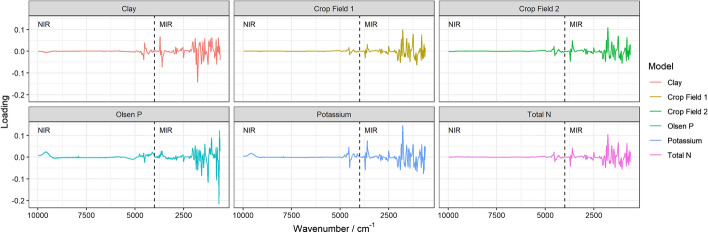
Loading values from the first component plotted as a function of wavenumber for PLSR models relevant in the modelling of lettuce diameter

As previously described, the loadings of the PLSR decomposition can be used for qualitative interpretation of the spectra. Peaks are caused by the soil property used as the response variable, whereas troughs indicate interference of different soil properties measured by the spectra. For each property included as a coefficient in the LMM, the loadings of the first component were plotted against wave number. The loadings of the first component in the final fitted PLSR models to predict lettuce diameter from the soil spectra were also plotted. The loadings from the total N PLSR model and to some extent those of the K^+^ PLSR model align better than the other soil properties with the loadings from the models that predict lettuce diameter from the soil spectra (Fig. 8). Closer loading alignment indicates that the same wave numbers explain an equal amount of variance from the response variable.

## Discussion

The aim of this study was to discover whether soil spectral measurements could be used to predict variation in the diameter of lettuce grown in commercial fields, both directly from the soil spectra and via predictions of soil properties.

### Prediction of lettuce diameter directly from the soil spectra

For both fields, the predictions of lettuce diameter from the soil spectra were close to observed values. These predictions were even better when separate models were fitted to each variety (top 4 panels Fig. 2). As the varieties were grown in blocks, the observed effect of variety could also be an effect of management that is independent of the soil. For example, some parts in Field 2 were not covered by the irrigation system. Furthermore, varieties associated with parts of the field with larger variation in soil properties were better predicted by the soil spectra (see concordance correlation coefficients in Tables 1, 2). Wetterlind et al. (2008) found that plant N uptake could only be modelled accurately for fields that showed a large range in soil organic matter and texture. The fields in this study are characterized by large variation in soil properties and hence can fulfil this requirement (Figs. S2 and S3).

### Precision of soil property predictions from soil spectra

Overall, the predictions for soil properties were good (Fig. 4). The reported errors might be optimistic for some properties because leave-one-out cross validation tends to over-estimate the accuracy and precision in PLSR (Viscarra Rossel 2008). The errors (expressed as root mean squares, RMSEs) proved to be similar to those found by other investigators: see Viscarra Rossel et al. (2016) for a review of 51 studies in which soil organic carbon was predicted from reflectance spectra with RMSEs ranging from 0.1 to 1.1%. However, the comparisons between results from this study and those reported in the literature need to be viewed with caution because the variances depend to some extent on the concentration of the target variable: smaller concentrations tend to be predicted with smaller errors. For example, Hutengs et al. (2018) reported RMSEs of 0.14–0.24% for SOC in the range 0.62–2.70%, whereas those from this study were 0.54% (from the leave-one-out cross validation) for the range 6.21–20.41%. Soil properties that had larger RMSE in their predictions are not spectrally active (e.g. S, P and K^+^). This accords with other studies in which the prediction of non-spectrally active properties are functions of their correlation with soil organic matter and particle-size distribution. Predictions for these properties are hence less robust than SOC and particle-size fractions (Du and Zhou 2009 and references therein). See Table S3 for the correlations between laboratory reference values.

The numbers of components included in the PLSRs are akin to those reported in the literature (namely from 3 to 9, Yang and Mouazen 2007; Wang et al. 2015; Hutengs et al. 2018). They rarely exceed 12, and they were fewer for soil properties that have a direct relation to molecular bonds in the MIR (e.g. C and clay). More components are generally retained for larger sets of spectral data where there are more pronounced differences in lithology, climate and other soil forming factors—see for example Dangal et al. (2019) and Lopo et al. (2016). The PLSR loadings from the first component indicate that the NIR region explains little of the variance and much less than those in the MIR region. This holds true for both the PLSR models to predict lettuce diameter and the models to predict soil properties. These findings accord with the literature. The loadings depend on the soil properties of interest and are unique for each study; nevertheless, the MIR region generally leads to more robust calibration than does the NIR (Viscarra Rossel et al. 2006; Yang and Mouazen 2007).

### Can values of soil properties predicted from the spectral data be used to predict lettuce diameter?

The LMs that related lettuce diameter from predicted soil properties performed reasonably well (MSE: 4.59 for Field 1 and MSE: 8.99 for Field 2). The prediction performance of the LM implies that it captures a large amount of the explanatory power of the IR spectra. However, the PLSR from the soil spectra alone predicted the lettuce diameter more precisely than did the LMs. It seems that the IR spectra capture more information about the soil relevant for crop growth than the soil properties included in the LM. This effect aligns with studies that compare crop predictions from IR spectra with crop predictions from laboratory reference values, in which the first outperforms the latter (Börjesson et al. 1999; Wetterlind et al. 2008).

The predictions from the LMMs (Eq. 6) were more precise than those from the soil spectra (by PLSR alone) with a difference in mean squared error (MSE) of 21 mm^2^ for Field 1 and of 51 mm^2^ for Field 2. This is because the LMMs account for the spatial structure in the lettuce diameters through the random term in the model. These results are somewhat misleading because the prediction of each lettuce relies on its spatial auto-correlation with the other lettuces in the field. In practice, growers would not be able to predict lettuce size at the beginning of the season with this model because they would not have these other measurements. Therefore, the auto-correlation in the model is not of practical use.

### Can soil spectra help growers with management?

This study showed two different ways of using the soil spectra. First, PLSR was used to predict lettuce diameter directly from the soil spectra. This does not allow for precise fertilizer or irrigation management as there is no information in how soil properties affect the lettuce response in the model. However, the predictions could be of practical use to management when the areas predicted to be high or low-yielding are consistent over seasons (which may not always be the case, Milne et al. 2012; Diacono et al. 2013 and references therein; Kindred and Sylvester-Bradley 2014) or if the grower has prior knowledge on why some areas are high or low-yielding. In this case, the management can be adapted based on an understanding of the causes of poor yields. For example, variable-rate planting or deciding not to crop certain areas if the soil spectra indicate likely poor yields that will lead to in-field yield waste. In this particular case, when lettuce are predicted to be less than 0.1 m in diameter (a size deemed too small by the growers) the grower may choose not to plant there.

The second approach was to predict lettuce diameter from estimated soil properties using a LM. Although predictions were poorer than the direct prediction by using the reflectance spectra, this approach gives growers more information and so could help them decide how to vary the application of fertilizer and irrigation within each field. These models are relevant when estimated soil properties are used for precise fertilization or irrigation. A remaining question is how to predict the exact amount of each nutrient needed by the crop at each place in the field and when to apply it (Baveye and Laba 2015; Kindred et al. 2017). The grower therefore needs prior knowledge on which soil properties influence crop growth for each specific part of each field. With this understanding, potential environmental impacts of farming can be minimised and profits maximised. This can be achieved by either not planting in low-yielding areas (and therefore no cost of fertilizer, herbicide and irrigation), or by managing inputs more precisely so that the economic return in crop response exceeds the amount spent on inputs. The LM and LMM reported here showed that total N, P, K and pH were significant predictors of lettuce size, indicating that variable rate application based on these properties could be used to advantage in lettuce production. Panagapoulos et al. (2006) demonstrated such an approach by creating “lettuce production capability” maps from kriged soil properties and identifying localised areas where the soil could be treated to improve yield. This illustrates the potential utility of predicting the variation of soil properties from IR spectra for the precision management of lettuce (in particular fertilizer and management of soil pH).

Soil spectra offer great promise for the precision management of crops but collecting the soil samples from the field and processing them (i.e. drying and milling) adds to the expense for a farmer. Therefore in practice, field-based spectral measurements are likely to be more attractive than spectral measurements made in the lab. Currently there has been limited exploration of field versus lab-based prediction errors with portable MIR spectrometers for soil properties other than soil carbon constituents (Ji et al. 2016a; Hutengs et al. 2019). Differences between field and lab-based MIR predictions range from 0 to 45% whereas the increase in error associated with VNIR techniques is reported to be as much as 57% (Ji et al. 2016a; Hutengs et al. 2019). Field based prediction for pH, organic matter and total nitrogen have been explored using (V)NIR with increases in prediction error of 14%, 27% and 22% compared to lab-based spectral measurements, respectively (Ji et al. 2016b). Most of the few existing studies on macro nutrient prediction using portable (V)NIR/MIR spectroscopy show limited success (Wenjun et al. 2014; Ji et al. 2016a). Poor prediction performance is commonly attributed to the absence of distinct features in the IR spectrum and varying relationships between total and available element content (Kuang et al. 2012; Pätzoldt et al. 2019). Consequently, the prediction models for available major and trace nutrients from soil spectra often prove to be less robust than those developed for particle size fractions and soil organic carbon. An exception is the study by Mouazen and Kuang (2016) with an 18% error increase for field-based soil available phosphorus predictions compared to predictions from spectra measured in the laboratory. These accurate predictions can probably be attributed to the large number of calibration samples used by the authors. Given that the linear models showed K and P to be important predictors of lettuce diameter, and that most studies show poor prediction of these variables from field-based measurements, further development of sensor technology is required for field-based measurements to be of practical use for this study’s methodology.

In the study reported here, properties of 30 samples from each field were measured by wet chemistry and the values were used to calibrate the models. Samples of this size are more than a commercial grower could expect to take in fields of 10.5 and 18.2 ha. In practice, there will be a trade-off between the number of calibration samples, with their associated costs, and accuracy of the prediction. Optimization of the sampling design, sample processing and the number of replicate measurements are examples of other factors that affect the accuracy. Optimization of sampling design depends not only on the sizes of samples to provide reference data but also on good coverage of the conditions within the field (Ramirez-Lopez et al. 2019 and references therein) or parent material (Sila et al. 2016). Once errors in soil predictions have been properly estimated these must be propagated through to the predictions of crop response. Only when these are properly accounted for could one estimate the true value of measuring the soil spectra for precision application of fertilizer and irrigation (Ramirez-Lopez et al. 2019).

Thus, the relevance of using the soil spectra directly or via estimated soil properties will depend on the situation. This study showed that under optimal conditions, there is potential for associating crop response to soil reflectance spectra. This association can be made directly from the soil reflectance spectra or by a regression that uses soil property values estimated by reflectance spectra.

## Conclusion

Reflectance spectra from soil in the near- and mid-infrared range were related to the diameters of lettuce grown in two fields in the Fenland region of England. They led to reasonably precise predictions of lettuce diameter and therefore are of value to the grower. The partial least squares regression (PLSR) that used soil spectra as response variables showed a mean squared error (MSE) of 39 mm^2^ for Field 1 and 68.7 mm^2^ for Field 2. Predictions of lettuce diameter that used linear models with the PLSR estimated soil properties gave somewhat poorer results, with a difference in MSE for Field 1: 6.9 mm^2^ and Field 2: 21.2 mm^2^). Predictions from the linear mixed models were more precise than those from the raw spectra (by PLSR alone) with a difference in MSE of 21.2 mm^2^ for Field 1 and of 51 mm^2^ for Field 2.

The spectra were related strongly to soil properties that determine crop growth, specifically, nitrogen (measured as total N), available phosphorus (P), exchangeable potassium (K^+^), clay content and pH. Using the values of the soil properties estimated from the reflectance spectra to predict the sizes of the lettuce was somewhat less precise than direct prediction from the spectra. The advantage to the grower of the indirect prediction is the gain in knowledge about which soil properties are important. This enables the grower to adapt the management to the soil. Precise indirect prediction is only feasible with a suitable calibration dataset that captures the variability of the underlying soil.

## Electronic supplementary material

Below is the link to the electronic supplementary material.

Supplementary file1 (DOCX 1211 kb)
