# Supplementary material for: Predicting the growth of lettuce from soil infrared reflectance spectra: the potential for crop management
Source: Precis Agric. 2020 Aug 10;22(1):226–48. doi: 10.1007/s11119-020-09739-x (PMC7814485; doi:10.1007/s11119-020-09739-x)
Supplement: Supplementary file 1 — Supplementary file1 (DOCX 1211 kb) [file 11119_2020_9739_MOESM1_ESM.docx]

# Supplementary Material

**Journal:**

Precision Agriculture

**Article title:**

Predicting the growth of lettuce from soil infrared reflectance spectra: the potential for crop management

**Authors:**

T.S. Breure^1^ · A.E. Milne^1^ · R. Webster^1^ · S.M. Haefele^1^ · J.A. Hannam^2^ · S. Moreno-Rojas^3^ · R. Corstanje^2^

Corresponding author: T.S. Breure

[timo.breure@rothamsted.ac.uk](mailto:timo.breure@rothamsted.ac.uk)

Telephone: +44 (0) 1582 763 133

^1^ Rothamsted Research, Harpenden AL5 2JQ, Great Britain

^2^ Cranfield University, Cranfield, Bedfordshire MK43 0AL, Great Britain

^3^ G’s Growers Ltd, Ely CB7 5TZ, Great Britain


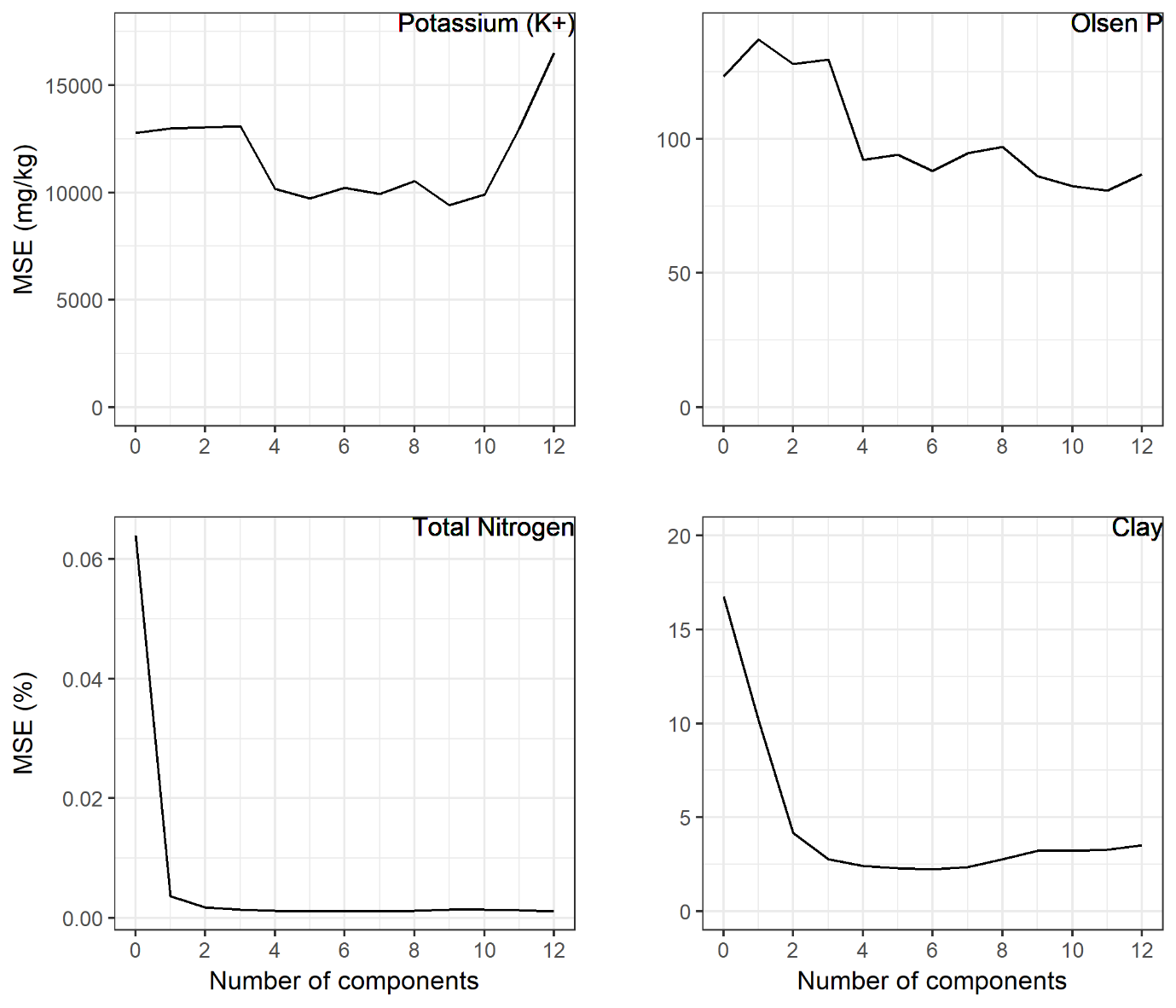


**Fig. S1.** Mean squared error (MSE) as a function of the number of components included within the partial least squares regression (PLSR) model in a leave-one-out (LOO) cross-validation procedure. Four examples of soil properties illustrate the numbers of optimum components for each soil property.


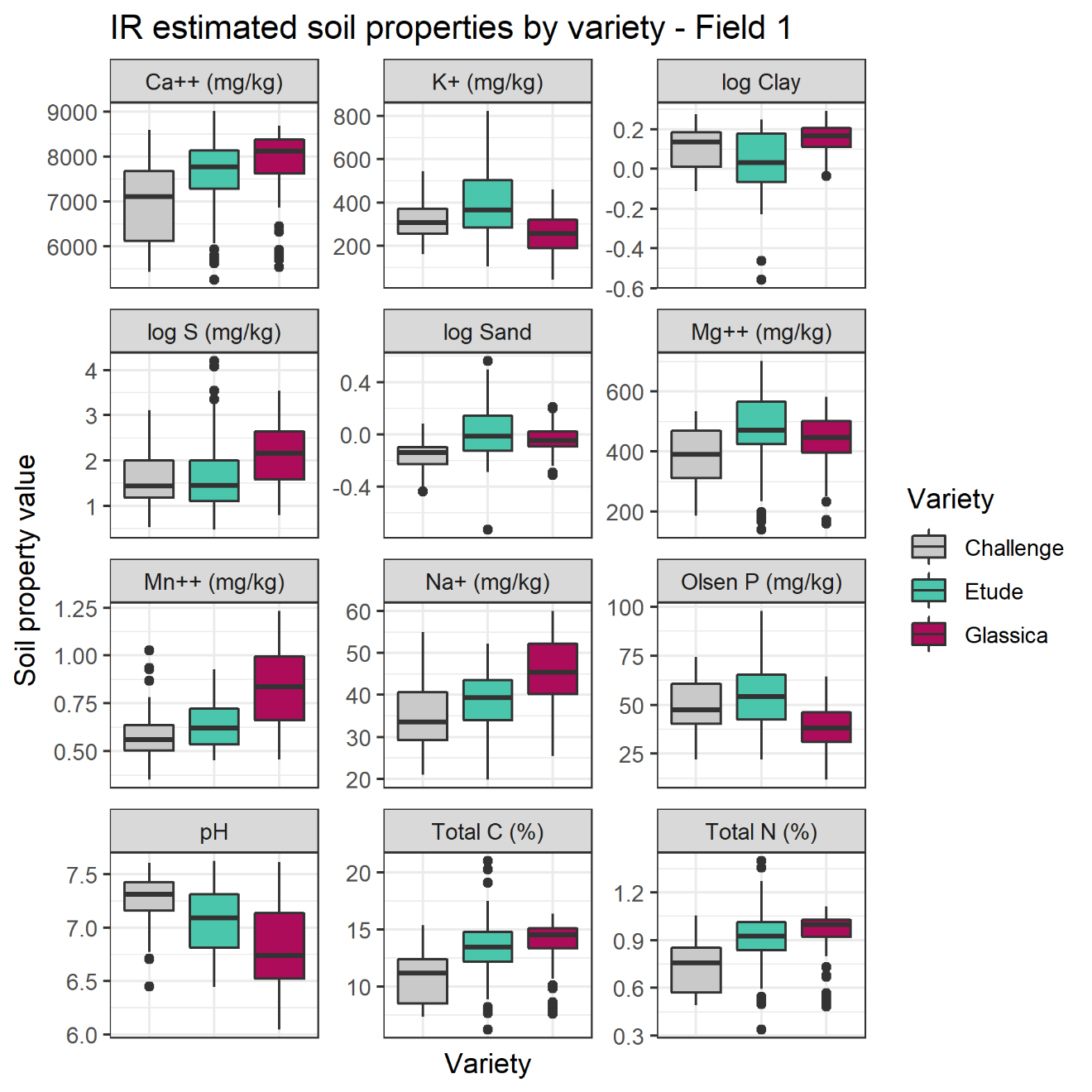


**Fig. S2.** Range of estimated soil properties by lettuce variety for Field 1. Soil properties were estimated from Near and mid-infrared (IR) spectroscopy measurements by partial least squares regression.


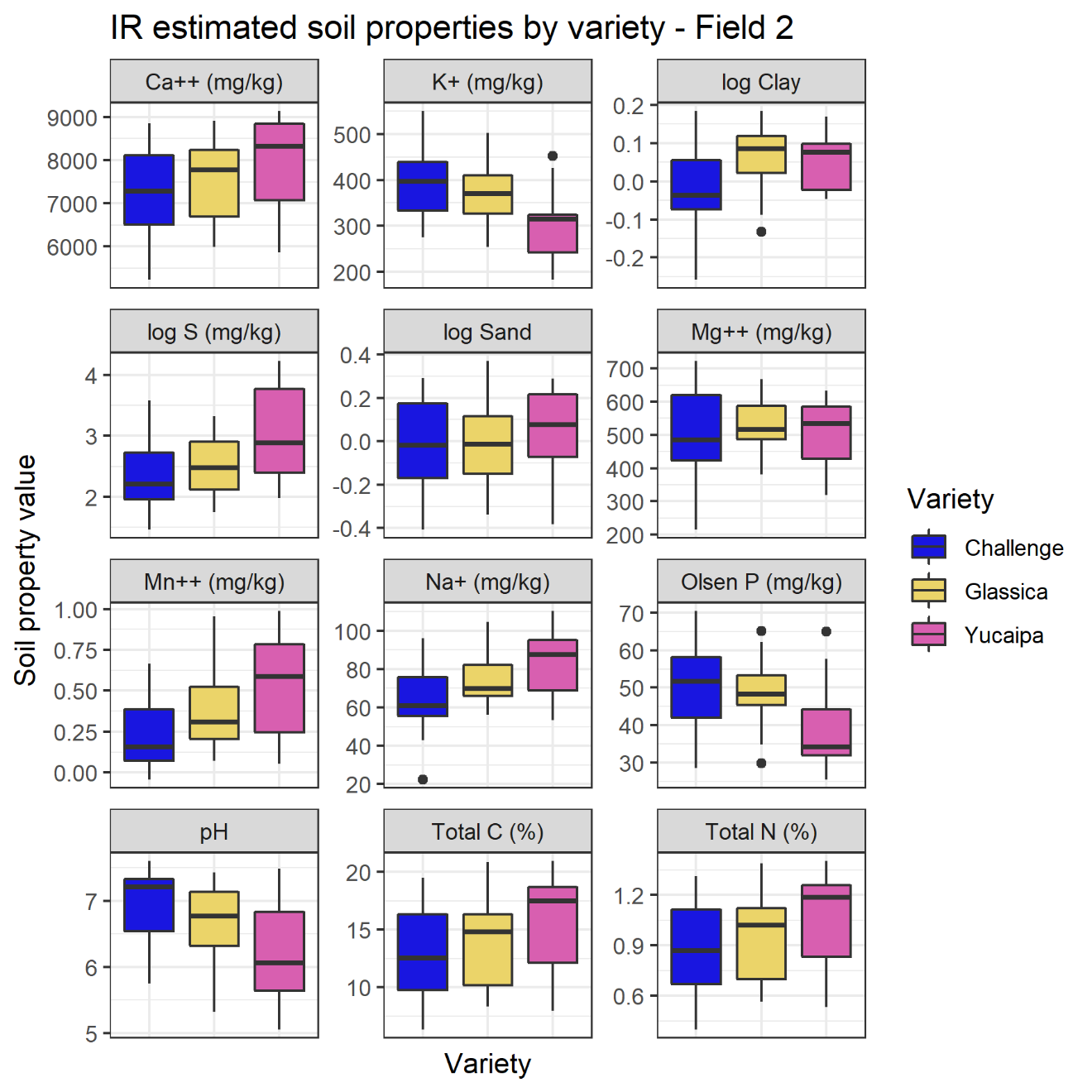


**Fig. S3.** Range of estimated soil properties by lettuce variety for Field 1. Soil properties were estimated from Near and mid-infrared (IR) spectroscopy measurements by partial least squares regression.

**Table S1**. Linear model (LM) and linear mixed model (LMM) fixed effects coefficients for Field 1

|  | Coefficient | |
| --- | --- | --- |
| Property | LM | LMM |
| Intercept | −29.1 | 8.44 |
| Total N/% | 8.42 | 2.40 |
| K^+^/mg kg^-1^ | 0.01 | 0.01 |
| $\log$(clay) | 5.63 | 7.57 |
| pH | 4.60 | NA |
| Variety Etude | 66.11 | −0.58 |
| Variety Glassica | 24.98 | −3.79 |
| Total N : Etude | −5.46 | 3.83 |
| Total N : Glassica | 7.88 | 10.67 |
| K^+^ : Etude | −0.01 | −0.01 |
| K^+^ : Glassica | 0 | −0.01 |
| $\log$(clay) : Etude | −17.7 | −20.4 |
| $\log$(clay) : Glassica | −16.19 | −15.20 |
| pH : Etude | 8.38 | NA |
| pH : Glassica | −3.78 | NA |

**Table S2.** Linear model (LM) and linear mixed model (LMM) fixed effects coefficients for Field 2

|  | Coefficient | |
| --- | --- | --- |
| Property | LM | LMM |
| Intercept | 8.71 | 8.47 |
| Total N/% | 10.24 | 9.56 |
| K^+^/mg kg^-1^ | 0.01 | 0.01 |
| P/mg kg^-1^ | −0.04 | 0 |
| Variety Glassica | −0.52 | 0.29 |
| Variety Yucaipa | 4.08 | 0.16 |
| Total N : Glassica | −0.61 | −0.43 |
| Total N : Yucaipa | −3.40 | −2.29 |
| K^+^ : Glassica | 0 | 0 |
| K^+^ : Yucaipa | 0.08 | 0.09 |
| P : Glassica | 0.02 | 0 |
| P : Yucaipa | −0.47 | −0.51 |

**Table S3.** Correlation between soil properties (n = 60) used to regress against near- and mid-infrared reflectance spectra by partial least squares methods.

| Property | Total C  % | Total N  % | Ca^2+^  mg kg^-1^ | K^+^  mg kg^-1^ | Mg^2+^  mg kg^-1^ | Na^+^  mg kg^-1^ | Mn^2+^  mg kg^-1^ | P  mg kg^-1^ | S  mg kg^-1^ | pH | Sand  % | | Clay  % | Silt  % |
| --- | --- | --- | --- | --- | --- | --- | --- | --- | --- | --- | --- | --- | --- | --- |
| Total C/% | 1 | 1 | 0.89 | 0.14 | 0.74 | 0.55 | 0.44 | 0.02 | 0.56 | -0.83 | | 0.73 | -0.51 | -0.75 |
| Total N/% | 1 | 1 | 0.9 | 0.14 | 0.74 | 0.55 | 0.44 | 0.02 | 0.57 | -0.83 | | 0.71 | -0.49 | -0.75 |
| Ca^2+^/mg kg^-1^ | 0.89 | 0.9 | 1 | 0.08 | 0.64 | 0.36 | 0.45 | -0.01 | 0.61 | -0.68 | | 0.52 | -0.26 | -0.74 |
| K^+^/mg kg^-1^ | 0.14 | 0.14 | 0.08 | 1 | 0.42 | 0.22 | -0.39 | 0.84 | -0.02 | 0.04 | | 0.12 | -0.21 | 0.11 |
| Mg^2+^/mg kg^-1^ | 0.74 | 0.74 | 0.64 | 0.42 | 1 | 0.76 | -0.03 | 0.22 | 0.5 | -0.59 | | 0.45 | -0.35 | -0.4 |
| Na^+^/mg kg^-1^ | 0.55 | 0.55 | 0.36 | 0.22 | 0.76 | 1 | -0.16 | 0 | 0.62 | -0.72 | | 0.44 | -0.39 | -0.31 |
| Mn^2+^/mg kg^-1^ | 0.44 | 0.44 | 0.45 | -0.39 | -0.03 | -0.16 | 1 | -0.44 | -0.01 | -0.42 | | 0.26 | -0.06 | -0.5 |
| P/mg kg^-1^ | 0.02 | 0.02 | -0.01 | 0.84 | 0.22 | 0 | -0.44 | 1 | -0.17 | 0.27 | | 0.08 | -0.16 | 0.1 |
| S/mg kg^-1^ | 0.56 | 0.57 | 0.61 | -0.02 | 0.5 | 0.62 | -0.01 | -0.17 | 1 | -0.7 | | 0.3 | -0.12 | -0.46 |
| pH | -0.83 | -0.83 | -0.68 | 0.04 | -0.59 | -0.72 | -0.42 | 0.27 | -0.7 | 1 | | -0.69 | 0.5 | 0.69 |
| Sand/% | 0.73 | 0.71 | 0.52 | 0.12 | 0.45 | 0.44 | 0.26 | 0.08 | 0.3 | -0.69 | | 1 | -0.91 | -0.65 |
| Clay/% | -0.51 | -0.49 | -0.26 | -0.21 | -0.35 | -0.39 | -0.06 | -0.16 | -0.12 | 0.5 | | -0.91 | 1 | 0.28 |
| Silt/% | -0.75 | -0.75 | -0.74 | 0.11 | -0.4 | -0.31 | -0.5 | 0.1 | -0.46 | 0.69 | | -0.65 | 0.28 | 1 |
